# Endovascular surgery for thoracic aortic pathologies involving the aortic arch

**DOI:** 10.3389/fcvm.2022.927592

**Published:** 2022-07-14

**Authors:** Heng Lu, Ling-chen Huang, Liang-wan Chen

**Affiliations:** ^1^Department of Cardiovascular Surgery, Union Hospital, Fujian Medical University, Fuzhou, China; ^2^Key Laboratory of Cardio-Thoracic Surgery, Fujian Medical University, Fujian, China

**Keywords:** aortic arch pathology, endovascular surgery, TEVAR, *in situ* fenestration, aortic arch reconstruction

## Abstract

**Background:**

Aortic arch pathologies are serious clinical conditions associated with a very dismal prognosis. Traditional open surgery has a high mortality and is not suitable for critically ill patients. Recently years, endovascular treatment of thoracic aorta has made rapid progress and has been gradually applied to the treatment of aortic arch pathologies. However, maintaining cerebral blood flow during endovascular treatment of aortic arch lesions remains a challenge at this time. This study aims to evaluate the feasibility, efficacy, and safety of endovascular treatment of thoracic aortic pathologies involving the aortic arch, and to present initial experience with this technique.

**Methods:**

From October 2016 to December 2020, patients who met the inclusion criteria were enrolled. All patients underwent thoracic endovascular aortic repair with the proximal landing zone of the stent-graft in the aortic arch at Ishimaru zones 0–1, in which cerebral flow needs to be maintained during surgery, and the supra-aortic branches were reconstruction with either *in situ* fenestration or the chimney technique.

**Results:**

A total of 62 cases with lesions involving the arch were treated with endovascular surgery. Total supra-aortic branches reconstruction was successfully performed in 51 cases, the left carotid artery (LCA) and the innominate artery reconstruction were performed in eight cases, the left subclavian artery (LSA) and the LCA were reconstructed in three patients. Among them, the *in situ* fenestration or chimney repair technique for the LSA was successful performed in 42 and 12 cases. However, in 20 patients, attempts to reconstruction the LSA using the fenestration technique were unsuccessful due to tortuous and angulated vessels. Early mortality was 6.45%. No neurological complications related to surgery occurred. Computer tomography images at post-operative follow-up (mean 3.51 months) confirmed patency of all branch stents without any signs of endoleaks, migration, conversion to retrograde dissection or receive open-heart surgery.

**Conclusion:**

The endovascular technique is an effective, feasible, safe and repeatable method to reconstruct the aortic arch, which allows for the reconstruction of the supra-aortic branches.

## Introduction

Aortic aneurysms and aortic dissection that involve the aortic arch are associated with extremely high mortality and morbidity. Untreated patients with acute type A aortic dissection have a mortality rate of 1–2% per hour on the first day, and nearly half die within a week (1, 2).

Traditionally, aortic aneurysms and aortic dissection that involve the aortic arch are repaired by open surgery to prevent aortic rupture or death from cardiac tamponade. The purpose of surgical repair is not only to redirect blood flow to the true lumen and eliminated the false lumen, but also to minimize brain damage during deep hypothermia with circulatory arrest. However, open surgery for aortic arch pathologies has been reported to have a 2–6% risk of mortality and a 2–7% risk of stroke. Emergency surgery even had a higher mortality (15%) and stroke rate (14%) (3).

However, there is a negative attitude toward surgical treatment of aortic arch pathologies in high-risk patients. Patients in poor physical condition, elderly adults and patients with impaired cardiac function or organ dysfunction are considered to be unable to tolerate open surgery. According to IRAD Registry, over 20% of patients with acute type A aortic dissection are denied open surgery because they are considered as high-risk patients for open surgery with circulatory hypothermic arrest (4).

With the advent of endovascular interventions, due to its minimally invasive characteristics, endovascular interventions are desirable to be used for high-risk patients. Although using endovascular therapy for aneurysm and aortic dissection is subject to many factors such as coronary artery disease, aortic valve disease, small diameter femoral access and shortage of proximal landing zone, a study of CT imaging suggested that approximately one third of those with type A aortic dissection may be suitable for endovascular repair (5). The difficulty of endovascular aortic arch reconstruction is to maintain cerebral flow during reconstruction of the left carotid artery (LCA) and the innominate artery (IA).

In recent decades, the thoracic endovascular aortic repair (TEVAR) has been a rapidly developing field worldwide. Initially used for the treatment of thoracic aortic aneurysms, TEVAR has been developed for the treatment of a variety of aortic lesions and is being explored for endovascular surgery of ascending aorta and arch lesions (6, 7).

Compared to open surgery, endovascular surgery offers the advantages of less surgical trauma, reduced mechanical circulatory support, and avoidance of aortic cross-clamping, thereby reducing cerebral complications, early mortality, and length of hospital stay (8–10). Therefore, in elderly or patients in poor physical condition who cannot tolerate cardiopulmonary bypass and hypothermia, after detailed pre-operative evaluation, confirm that the patient does not have aortic regurgitation or coronary artery disease, endovascular treatment can be provided as an appropriate treatment options to minimize surgical injury in these high-risk patients (11).

Nowadays, following the development of endovascular techniques, Ishimaru zones were introduced as a new classification to divide the aorta into multiple regions. In an expert consensus focused on the treatment of thoracic aortic diseases, especially lesions involving the aortic arch, the Ishimaru classification was used to segment the aorta according to the landing zone of the proximal and distal attachments (12, 13).

In this study, we retrospectively analyzed the clinical outcomes of patients with aortic arch pathologies who underwent endovascular repair with stents landing proximally in zones 0–1 between October 2016 and January 2020. The results of patients’ follow-up were also collected and evaluated. The aim of our study was to evaluate the safety, efficacy, and feasibility of endovascular repair of thoracic aortic pathologies involving the aortic arch, and to present initial experience with this technique.

## Materials and methods

### Patients and methods

This was a single-center retrospective study that included 62 patients with aortic lesions involving supra-aortic branches who underwent endovascular repair at our institution from October 2016 to December 2020. All patients enrolled for endovascular aortic arch repair were evaluated by a multidisciplinary team and deemed unsuitable for open-heart surgery. The reason for selecting a particular patient for endovascular surgery included elderly patients, previous history of cardiac surgery or patients with poor physical function or severe comorbidities. The inclusion criteria were Stanford type A aortic dissection, retrograde type A aortic dissection, intramural hematoma, aortic ulcer, and aneurysm of the aortic arch which involving the orifice of supra-aortic branches. In other word, the proximal landing zone need to cover the Ishimaru zones 0–1. The criteria for exclusion were as follows: (1) patients with severe peripheral vascular diseases or small-diameter femoral artery access; (2) the diameter of the aorta from the proximal to the distal landing zone is greater than 40 mm (or the maximum stent size cannot be able to fully seal the aneurysm); (3) the patient with aortic dissection was comatose or complicated with irreversible abdominal visceral ischemia; and (4) severe coronary artery or aortic valve disease.

For each patient who underwent endovascular repair of aortic arch, the computed tomographic angiography (CTA) data and digital subtraction angiography (DSA) imaging were taken from our hospital’s picture achieving and communication system. Discharge summaries, operative notes and post-operative outcome data were abstracted from the electronic medical record system.

All the related clinical data were documented, and surgical strategies, surgical complications and overall clinical outcomes were noted. We were particularly concerned about stroke, endoleak, migration of grafts, retrograde aortic dissection, paraplegia, and death – complications that may be related to the endovascular surgery.

The ethics committee of Union Hospital of Fujian Medical University approved this study protocol (Ethical approval number: 2022KY100), the requirement for informed consent was waived due to the retrospective nature of this research.

### Surgical techniques

In our institution, the endovascular surgery was performed by cardiovascular surgeons with extensive experience and mature surgical skills. Nowadays, the endovascular treatment of the aortic arch pathologies is still formidable and challenging because it is influenced by the diameter, angle, and elasticity of the aortic arch. In addition, for endovascular reconstruction of the aortic arch and supra-aortic branches, the cerebral protection techniques reported in the literature are highly variable. Some centers use cardiopulmonary bypass for intraoperative brain protection, while others even use extracorporeal membrane oxygenation for brain protection.

In our center, with the time required to reconstruct the supra-aortic branches gradually decreases as technical proficiency increases, our surgical steps can therefore be divided into two stages. First stage, under pressure monitoring, after the release of the endovascular aortic stent, temporary femoral artery-common carotid artery bypass was initiated to maintain the cerebral blood flow while reconstruct the LCA and the IA. With the proficiency of the operating technique, instead of femoral artery-common carotid artery bypass, the sheath was first inserted into the ascending aorta through the right common carotid artery, and the sheath was used to form a gutter space between the periphery of the sheath and the main stent (monitoring intraoperative cerebral oxygen and blood pressure of the right upper limb). The gutter space can also supply blood to the brain, meanwhile the blood flow of the LCA can be quickly reconstructed (in most cases, the origin and course of the left common carotid artery is almost perpendicular to the aortic arch, so it is easier to achieve fenestration). And then retracted the sheath and fenestration the IA.

**FIGURE 1:**
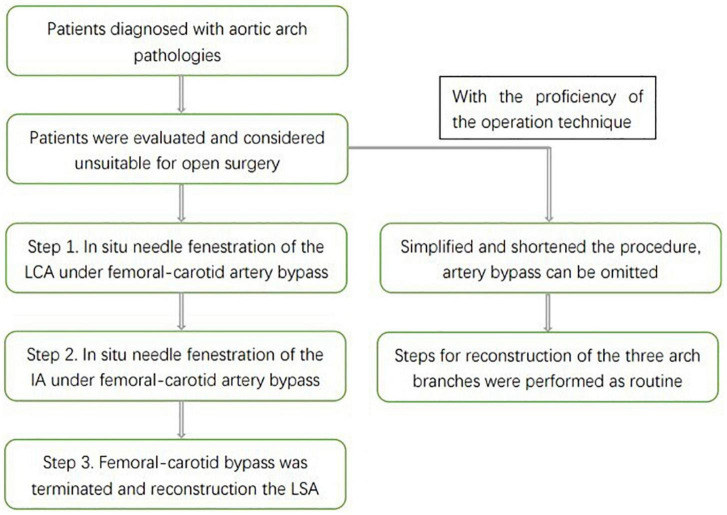
A schematic drawing that illustrates the intervention steps of endovascular aortic arch repair.

The endovascular total aortic arch reconstruction is used as an example to briefly introduce the basic steps of the procedure and the measures for brain protection in our center (Figure 1).

**FIGURE 2:**
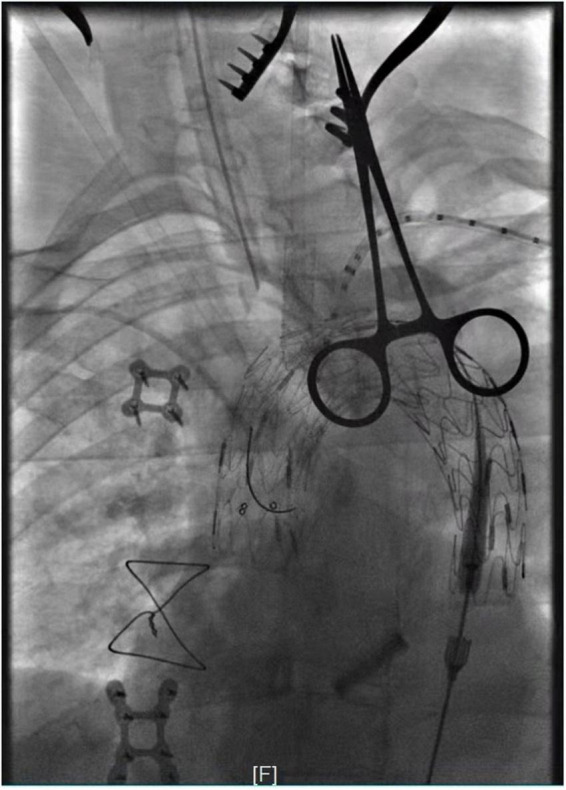
Intra-operative angiography after *in situ* fenestration of the LCA, it was the first branch of the aortic arch to undergo *in situ* fenestration.

Under general anesthesia, the patient was placed in the supine position, and the bilateral inguinal region, bilateral neck and left upper extremity were disinfected. Then incised to expose the bilateral femoral arteries, bilateral common carotid arteries and left brachial artery. The left brachial artery was accessed with an 8-Fr short sheath, then a 5-Fr pigtail catheter was inserted and the catheter tip was placed in the aortic root for angiography. The location of the tears, the extent of lesion involvement, the diameter of the aortic arch and the dominant vertebral artery were determined by angiography and compared with pre-operative CTA to check for new lesion development. Then we were about to release the stent and reconstruct the supra-aortic branches. A temporary femoral artery-common carotid artery bypass was established, the perfusion tip should not exceed the position of the carotid bifurcation, and the bridge pressure was monitored during the operation. The invasive arterial pressure of the bypass bridge was measured to ensure that it was greater than 60 mmHg, which would ensure a longer time for *in situ* fenestration. The 16 Fr sheath was inserted into the ascending aorta by retrograde puncture of the right common carotid artery. Endovascular aortic arch repair was then performed, the Ankura™ I/II Thoracic Stent Graft System (Lifetech Scientific, Shenzhen, China) was completely covered the aortic arch lesion from the ascending aorta to the distal descending aorta. During this period, the cerebral blood flow is supplied by the femoral artery *via* the femoral-carotid artery bypass. At the same time, the periphery of the sheath and the main stent form a gutter space, which can also supply blood to the brain. It forms a double brain protection, which can ensure the safety of the operation. Then *in situ* fenestration of the LCA was performed (Figure 2). As mentioned earlier, this artery runs straight and is easy to perform *in situ* fenestration, allowing for rapid stenting of the LCA. The we performed *in situ* fenestration with a hollow live biopsy needle, the sheath should be in contact with the expanded polytetrafluoroethylene (e-PTFE) fabric-covered of the stent-graft. The hollow-needle was introduced *via* the sheath, then directly contacted the fabric-covered of the stent-graft, and the *in situ* fenestration is applied as vertically as possible. After creation of the proximal fenestration and introduction of the hollow needle into the stent graft, a hydrophilic 0.035-inch Stiff guidewire was switched with a balloon catheter that was advanced through the fabric covering into the stent. Following balloon dilatation, a covered short stent graft was deployed in the fenestration and the patency of the endograft fenestration was assessed by DSA.

**FIGURE 3:**
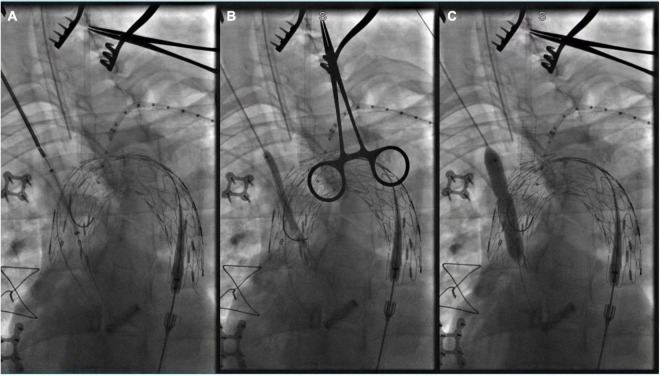
Panel **(A–C)** showed *in situ* fenestration of the IA made by gradual balloon dilation (after the fabric part of the main stent-graft was punctured by using a hollow needle).

**FIGURE 4:**
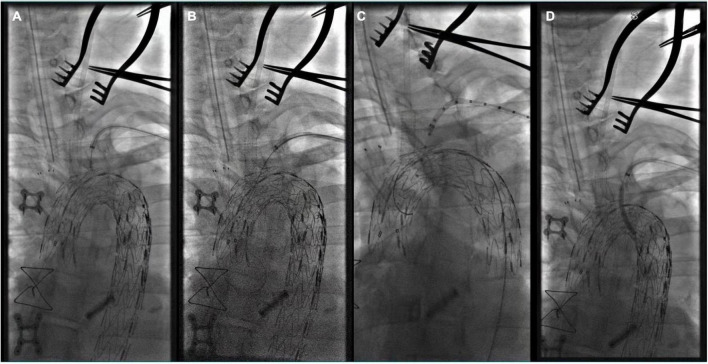
**(A)** The tip of Fustar sheath was deflected and directly contact the fabric part of the main stent graft and *in situ* needle fenestration was applied; **(B)** 0.018 inch guidewire was punctured into the main stent; **(C)** A pigtail angiographic catheter was exchanged and advanced into the ascending aorta for angiography; **(D)** The fenestration was dilated by the balloon catheter after successful fenestration in the LSA.

**FIGURE 5:**
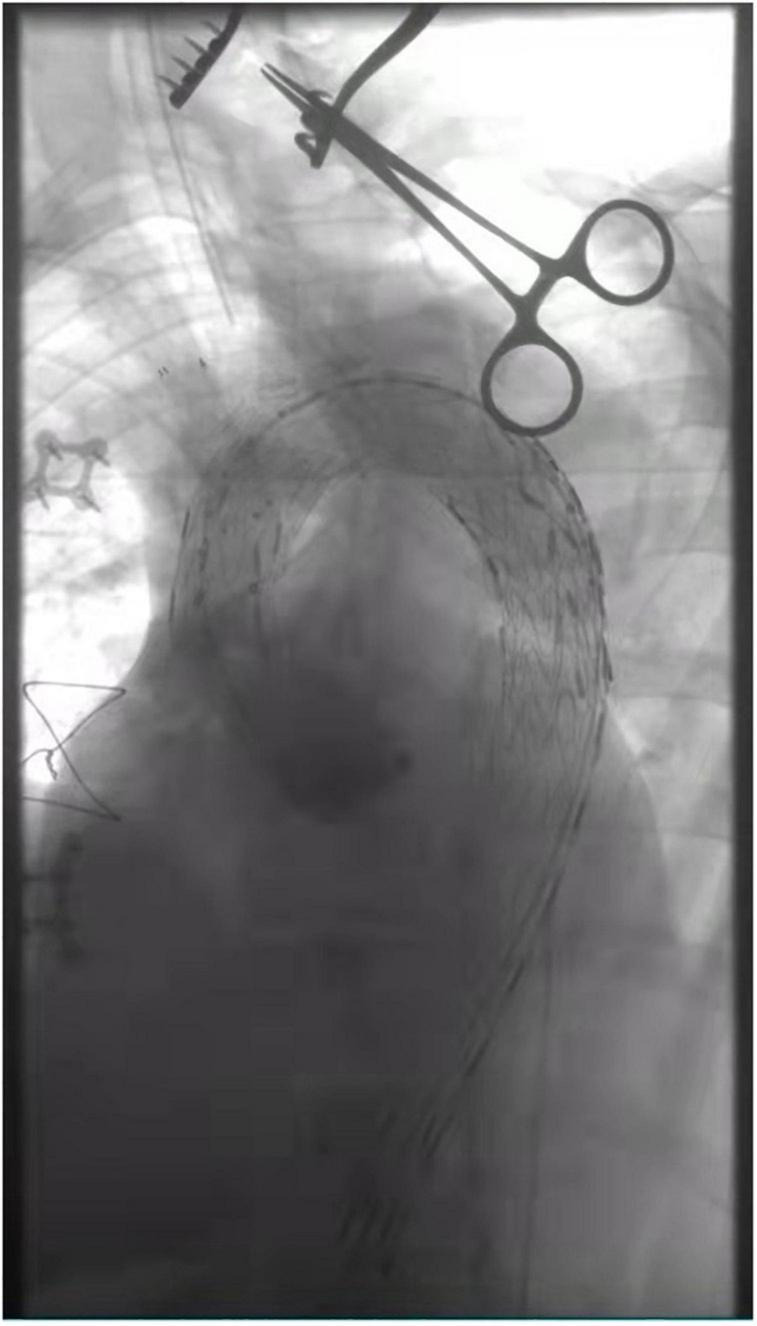
The three branches of the aortic arch were well displayed, and the IA, LCA, and LSA were reconstructed with *in situ* fenestration.

After rapid establishment of blood flow to the LCA, the IA was fenestrated *in situ* in the same way (Figure 3). After stenting of the IA, the temporary bypass was removed. Then we perform the left subclavian artery (LSA) *in situ* fenestration. We used the Fustar™ Steerable Introducer system (Lifetech Scientific, Shenzhen, China) for LSA *in situ* fenestration. It was a combination of long sheath and guiding catheter which the tip can be deflected. After introduced the Fustar sheath *via* the LSA, the tip was deflected in order to directly contact the fabric part of the main stent graft and *in situ* needle fenestration was applied. After the 0.018 guidewire penetrated into the stent graft, a balloon catheter was advanced inside the stent graft *via* the fabric covered of the stent graft and then dilated gradual by different sizes of balloons. Following dilatation of the balloon, a covered stent graft was implanted in the fenestration and DSA was applied to verify the patency of the endograft fenestration (Figure 4). During this period, if *in situ* fenestration is difficult to perform, the chimney technique is an option. The LSA would be occluded to prevent endoleak if not successful reconstruction the LSA and if the aneurysm cavity is large and endoleak is severe, the candy-plug technique or coil embolization can still be used for false lumen occlusion (14). After totally reconstruction of the supra-aortic branches, angiography was performed to confirm that there were no intra-operative problems, and the surgery was terminated (Figure 5).

With the proficiency of the operation technique, the femoral-carotid artery bypass can be omitted with monitoring of cerebral oxygen and right radial artery pressure, and other steps were performed as routine.

### Clinical parameters definition

In this study, death within 30 days after surgery was considered early mortality. Stroke was defined as cerebral infarction or intracerebral hemorrhage. Kidney Disease Improving Global Outcomes (KDIGO) criteria (15) were used to define acute kidney injury. Endoleaks were defined as persistent blood flow outside the graft and within the aneurysm sac (16).

### Follow-up

Routine post-operative follow-up imaging with CTA was performed at 1 week, 3 months after surgery to evaluate endoleak, migration, and the patency of supra-aortic branches, as well as morphology of dissection or aneurysm with remodeling.

### Statistical analysis

Statistical analysis was performed with SPSS ver. 21.0 (SPSS Inc., Chicago, IL, United States). Normally distributed continuous variables were expressed as mean with standard deviation; non-normally distributed continuous variables were expressed as median and interquartile range.

## Results

### Baseline data

From October 2016 to December 2020, 62 patients received treatment for different types of aortic arch lesions. All patients were carefully selected to receive TEVAR and to undergo revascularization of the supra-aortic vessels during TEVAR.

There were 39 males and 23 females. The mean age of the study population was 65.16 ± 10.27 years (range 45–83 years). The mean BMI values of the patients were 25.21 ± 2.02 kg/m^2^. There were 49 patients (79.03%) with hypertension and 16 patients (25.81%) with diabetes. Twenty-two had a history of smoking, nine had peripheral vascular disease, only one had pre-operative paraplegia, and seven had renal insufficiency.

For the specific type of aortic dissection, 15 patients were diagnosed with retrograde type A aortic dissection, 12 were diagnosed with type A aortic dissection, 5 with penetrating aortic ulcer, 23 had symptomatic large thoracic aortic aneurysms, and 7 had intramural hematomas. All Demographic and clinic data are shown in Table 1.

**TABLE 1:** Demographic data.

Item	Data
Female/male	23/39
Age (years)	65.16 ± 10.27
BMI (kg/m^2^)	25.21 ± 2.02
Smoking (*n*, %)	22 (35.48%)
Hypertension (*n*, %)	49 (79.03%)
Diabetes (*n*, %)	16 (25.81%)
Peripheral vascular disease (*n*, %)	9 (14.52%)
Renal insufficiency (*n*, %)	7 (11.29%)
Paraplegia (*n*, %)	1 (1.61%)
**Type of pathologies (*n*, %)**	
Retrograde type A aortic dissection	15 (24.19%)
Type A aortic dissection	12 (19.35%)
Intramural hematoma	7 (11.29%)
Aortic ulcer	5 (8.06%)
Aortic arch aneurysm	23 (37.10%)

### Surgical detail

Thoracic endovascular aortic repair was technically successful performed in all 62 patients with lesions involving the arch. Total arch reconstruction was performed in 51 cases, IA and LCA reconstruction was performed in 8 cases. LCA and LSA reconstruction in 3 patients. Among them, LSA *in situ* fenestration and chimney techniques were successful in 42 and 12 cases, respectively. Reconstruction of three supra-aortic branches in 51 patients were successfully performed, but attempts to perform *in situ* LSA fenestration in 20 patients were unsuccessful due to the tortuosity of the LSA and the formation of an acute angle between the LSA and the aortic arch. All surgical data are shown in **Table 2**.

### Early outcome

The mean post-operative mechanical ventilation time for the patients was 13.11 ± 3.10 h. The intensive care unit length of stay was 39.91 ± 10.16 h. The length of hospital stay ranged from 6 to 31 days, with a mean of 9.55 ± 3.61 days. There were four (6.45%) early deaths. One patient died during the operation. One patient suffered an ischemic stroke and died on post-operative day 3. Another patient died on post-operative day 21 from severe pneumonia and secondary sepsis. The last patient died of multiple organ failure on the 31st post-operative day. No fenestration related neurological complications occurred. Two patients (3.23%) developed permanent neurological deficits. Ischemic stroke was diagnosed in both of these 2 patients. Three patients experienced post-operative haemodialysis-dependent renal failure (4.84%). One patient suffered with pre-operative paralysis of the lower limbs remained paralyzed after surgery. No post-operative malperfusion syndrome occurred.

### Follow up

The mean duration of early follow-up time was 3.51 months. No endoleak and migration of stents was discovered. False lumen thrombosis and subsequent positive remodeling of the aorta were evidenced, and all the reconstruction supra-aortic branches were confirmed to be patency by post-operative follow-up CTA imaging. All related post-operative complications and follow-up data are shown in **Table 3**.

## Discussion

Despite significant advances in surgical techniques, perfusion techniques, anesthesia and perioperative management in recent decades, total arch reconstruction is still the most difficult challenge in cardiovascular surgery. Due to the effects of hypothermia circulation arrest, the mortality and cerebral complications of traditional total arch replacement are quite high. This high-risk procedure was reported to have about 2–6 percent death risk and 2–7 percent stroke risk. The mortality rate and stroke rate were higher for emergency surgery, at 15 and 14%, respectively (3, 17–19).

When endovascular surgery is feasible, De Rango et al. showed that endovascular surgery can reduce mortality and complication rates compared to open arch procedure. In their study, patients with open aortic arch repair had a 30-day mortality rate of 13.8% compared with 8.5% in patients with endovascular aortic arch reconstruction, despite the fact that patients with endovascular treatment were older, more severe, and had more comorbidities (11). Another recent study also supports the low mortality and cerebral complications associated with endovascular total aortic arch repair (20). Therefore, for patients who are in poor systemic conditions and cannot tolerate open surgery, endovascular total aortic arch repair seems to be an attractive alternative.

Endovascular aortic stent grafting was originally designed as an alternative technique to repair abdominal aortic aneurysms. In 1994, Dake et al. attempted to apply stent grafts to thoracic descending aortic aneurysms and reported the first successful TEVAR procedure (21). Although Dake demonstrated that TEVAR was repeatable and safe, the U.S. Food and Drug Administration did not approve TEVAR for human implantation until after the Gore TAG critical trial was completed in 2005. Back then, only descending thoracic aneurysms located in Ishimaru zone 3–5 were approved (12, 22). Customarily, the first requirement for TEVAR is to have a normal segment of aorta (≥ 1.5 cm) at each end of the aortic lesion as a landing zone. Clinically, the location of the tear in some patients is very close to the LCA, LSA, and IA, and may even expand to the ascending aorta. TEVAR is difficult to perform in these patients because the lesions are too close to the supra-aortic branches and there is not enough landing zone. But with the ongoing development of stent graft technology, the indications for active application of TEVAR in a variety of lesion types have expanded. However, it is considered to be “off-label” for these additional applications and there are few randomized trials comparing endovascular and open treatments (17). Currently, according to the 2010 guidelines, the FDA has not yet approved endovascular stents for the treatment of aneurysms or other diseases of the aortic arch (17). But in 2008 alone, a United States study showed that more than 50% of TEVAR applications were either not approved by the FDA or were hybrids (23).

Thoracic endovascular aortic repair is rapidly developing and being applied in an increasing number of aortic lesion sites and types. Considering the perioperative period, short- and mid-term incidence of mortality and morbidity, TEVAR is rapidly replacing conventional open surgery as it is a preferred approach for patients with descending aortic disease, and surgical approaches for lesions involving the ascending aorta and aortic arch are being explored. The search for minimally invasive methods to treat arch lesions is driven by one simple fact: endovascular grafts are particularly valuable for patients with significant complications (age, severe cardiac, pulmonary, and renal insufficiency), who have severe comorbidities that make them unsuitable candidates for open arch surgical repair.

Despite the promising early outcomes of endovascular aortic arch reconstruction, limited by the difficult to maintain cerebral blood flow during surgery and anatomical characteristics of aorta pathologies, only a few centers can perform this type of endovascular surgery. At present, endovascular aortic arch repair still faces a series of challenges. The main challenge is to cover the arch with a stent and maintain cerebral blood flow during this period, and to avoid intraoperative cerebral embolization. This article reviews the clinical management of patients who underwent endovascular procedures at our institution to assess the mortality rate, the incidence of cerebral complications, the incidence of poor perfusion syndrome, and the pros and cons of this technique.

In terms of mortality and incidence of cerebral complications, a comparative study of conventional open surgical repair and endovascular surgical reconstruction showed that endovascular surgery was a valid alternative to conventional open surgery in terms of surgical mortality and neurological events (11). In addition, although cerebrovascular accidents are a worrisome complication of open aortic arch repair, but studies showed that the early incidence of cerebrovascular accidents in endovascular aortic arch repair only ranging from 0 to 5.4% (24–26). In our study, using our standard surgical procedure and subsequent clinical outcomes, cerebral complications and mortality showed similar satisfied and encourage results.

Despite of the favorable outcome, in our clinic practice, there are still some problems may be encountered during total endovascular aortic arch repair, such as the anatomical variant of aortic arch, the orifice location of the LSA, bovine arch, gothic arch, giant arch aneurysm, shortage of proper surgical equipment, and inappropriate vascular access (severe bend in the aorta, lack of proper large diameter femoral access, iliac artery disease) (27). Any one of these factors may result in failure of stent delivery, incorrect positioning, migration of grafts and ultimately treatment failure. During we performed *in situ* fenestration the total endovascular aortic arch reconstruction surgery, based on our experience, we found that the difficulty of the surgery was mainly in the following aspects. For challenging aortic arch morphology such as “type III arch,” twisted LSA, and giant aortic arch aneurysm, certain strategies and techniques should be used for *in situ* fenestration.

In our opinion, *in situ* fenestration of “type III arch” is very difficult. Type III aortic arch is used to refer to that the IA originates below the horizontal plane of the inner curvature of the aortic arch (17). The key to successful fenestrate is to ensure that the puncture sheath tip stable against the membrane of the main body stent. Geometrically, the relationship between the tip of the puncture needle and the membrane of the main stent is most stable if the tip is vertical to the membrane. However, in “type III arch” or “steep arch,” the angle between the branches of the arch and the aorta is very small, and the angle between the needle and the main stent is also very small. When the needle tip is close to the membrane, the sheath tip will easily shift or slip off.

*In situ* fenestration is more difficult for huge aneurysms involving the arch. When the aneurysm cavity of the arch is huge, the stent is far away from the three branches of the arch after release. In this case, the sheath is almost floating in the aneurysm cavity, and it is more difficult to stabilize the sheath against the fabric part of stent graft. For such arch morphology where there is a large space between the supra-aortic branches and the main stent, a precise multi-angle DSA may have some help. Especially if the aneurysm cavity is very huge, our success rate is not very high.

In our practice, the anatomically variant of the LSA is the most difficult to fenestrate among the three branches of the aortic arch. The anatomy of the LSA is very variable, some of them have an abnormal twisted morphology, some of them have a very small angle with the arch, some of them are narrow-mouthed or are accompanied by atherosclerotic plaques, all these anatomical variations make it difficult for the tip of the sheath *via* the brachial artery to steadily contact on the fabric part of the stent graft when performed *in situ* fenestration. If the success of fenestration is far beyond reach, we can also choose to perform embolization of the LSA after assessing the vertebral artery advantage.

In conclusion, endovascular aortic arch repair is a surgical challenge, regardless of the technique used, whether fenestration or chimney. We report on the possibility of endovascular total aortic arch reconstruction with standard stent graft and reserve the brain vessels through *in situ* fenestration or chimney technique. Many people may be concerned about the risk of endoleaks and stent obstruction, but in our study, no stent blockage was observed at least 3 months of follow-up, and our study showed a low incidence of significant endoleaks after endovascular aortic arch repair.

Despite these encourage outcomes, however, there are still some limitations. There was no surgical control group and only a small number of patients were studied. Besides, long-term follow-up data are lacking, and there is no high-level evidence to recommend the routine use of endovascular techniques for the treatment of aortic arch pathologies. Although the trauma of open surgical repair is intensive for many elderly and weaker patients to bear, but the outcome of totally endovascular aortic repair is also questionable. A recent work confirmed that *in situ* fenestration causes substantial damage to the fabric covered of the stent graft (28).

## Conclusion

Endovascular aortic arch reconstruction using *in situ* fenestration or chimney technique is a safe, feasible, effective and repeatable method, which can achieve the reconstruction of the supra-aortic branches. However, the follow-up period should be extended to assess the durability of this technology.

## Data availability statement

The raw data supporting the conclusions of this article will be made available by the authors, without undue reservation.

## Ethics statement

The studies involving human participants were reviewed and approved by the Ethics Committee of Fujian Medical University. Written informed consent for participation was not required for this study in accordance with the national legislation and the institutional requirements.

## Author contributions

L-WC, L-CH, and HL designed the study, participated in the operation, and drafted the manuscript. L-CH and HL collected the clinical data and performed the statistical analysis. HL and L-WC provided the technical support. All authors read and approved the final manuscript.

## Conflict of interest

The authors declare that the research was conducted in the absence of any commercial or financial relationships that could be construed as a potential conflict of interest.

## Publisher’s note

All claims expressed in this article are solely those of the authors and do not necessarily represent those of their affiliated organizations, or those of the publisher, the editors and the reviewers. Any product that may be evaluated in this article, or claim that may be made by its manufacturer, is not guaranteed or endorsed by the publisher.

## Abbreviations

IA: innominate artery
LCA: left carotid artery
TEVAR: thoracic endovascular aortic repair
DSA: digital subtraction angiography
CTA: computed tomographic angiography
LSA: left subclavian artery.
